# Biosensor Real-Time Affective Analytics in Virtual and Mixed Reality Medical Education Serious Games: Cohort Study

**DOI:** 10.2196/17823

**Published:** 2020-09-02

**Authors:** Panagiotis E Antoniou, George Arfaras, Niki Pandria, Alkinoos Athanasiou, George Ntakakis, Emmanouil Babatsikos, Vasilis Nigdelis, Panagiotis Bamidis

**Affiliations:** 1 Lab of Medical Physics The Medical School Aristotle University of Thessaloniki Thessaloniki Greece

**Keywords:** virtual patients, affective learning, electroencephalography, medical education, virtual reality, wearable sensors, serious medical games

## Abstract

**Background:**

The role of emotion is crucial to the learning process, as it is linked to motivation, interest, and attention. Affective states are expressed in the brain and in overall biological activity. Biosignals, like heart rate (HR), electrodermal activity (EDA), and electroencephalography (EEG) are physiological expressions affected by emotional state. Analyzing these biosignal recordings can point to a person’s emotional state. Contemporary medical education has progressed extensively towards diverse learning resources using virtual reality (VR) and mixed reality (MR) applications.

**Objective:**

This paper aims to study the efficacy of wearable biosensors for affect detection in a learning process involving a serious game in the Microsoft HoloLens VR/MR platform.

**Methods:**

A wearable array of sensors recording HR, EDA, and EEG signals was deployed during 2 educational activities conducted by 11 participants of diverse educational level (undergraduate, postgraduate, and specialist neurosurgeon doctors). The first scenario was a conventional virtual patient case used for establishing the personal biosignal baselines for the participant. The second was a case in a VR/MR environment regarding neuroanatomy. The affective measures that we recorded were EEG (theta/beta ratio and alpha rhythm), HR, and EDA.

**Results:**

Results were recorded and aggregated across all 3 groups. Average EEG ratios of the virtual patient (VP) versus the MR serious game cases were recorded at 3.49 (SD 0.82) versus 3.23 (SD 0.94) for students, 2.59 (SD 0.96) versus 2.90 (SD 1.78) for neurosurgeons, and 2.33 (SD 0.26) versus 2.56 (SD 0.62) for postgraduate medical students. Average alpha rhythm of the VP versus the MR serious game cases were recorded at 7.77 (SD 1.62) μV versus 8.42 (SD 2.56) μV for students, 7.03 (SD 2.19) μV versus 7.15 (SD 1.86) μV for neurosurgeons, and 11.84 (SD 6.15) μV versus 9.55 (SD 3.12) μV for postgraduate medical students. Average HR of the VP versus the MR serious game cases were recorded at 87 (SD 13) versus 86 (SD 12) bpm for students, 81 (SD 7) versus 83 (SD 7) bpm for neurosurgeons, and 81 (SD 7) versus 77 (SD 6) bpm for postgraduate medical students. Average EDA of the VP versus the MR serious game cases were recorded at 1.198 (SD 1.467) μS versus 4.097 (SD 2.79) μS for students, 1.890 (SD 2.269) μS versus 5.407 (SD 5.391) μS for neurosurgeons, and 0.739 (SD 0.509) μS versus 2.498 (SD 1.72) μS for postgraduate medical students. The variations of these metrics have been correlated with existing theoretical interpretations regarding educationally relevant affective analytics, such as engagement and educational focus.

**Conclusions:**

These results demonstrate that this novel sensor configuration can lead to credible affective state detection and can be used in platforms like intelligent tutoring systems for providing real-time, evidence-based, affective learning analytics using VR/MR-deployed medical education resources.

## Introduction

### Affective Learning

According to Bloom’s taxonomy of learning domains, there are three main domains of learning, namely cognitive (thinking), affective (emotion/feeling), and psychomotor (physical/kinesthetic) [1]. Specifically, in the affective domain, learning objectives focus on the learner’s interests, feelings, emotions, perceptions, attitudes, tones, aspirations, and degree of acceptance or rejection of instructional content [2]. Despite the fact that defining emotion is a rather daunting process, the term has been defined as “an episode of interrelated, synchronized changes in the states of all or most of the five organismic subsystems in response to the evaluation of an external or internal stimulus event as relevant to major concerns of the organism” [3]. Furthermore, emotion has been defined from a psychological stance as a conscious representation of what individuals feel, whereas from a neuropsychological point of view, emotion “is seen as a set of coordinated responses that take place when an individual faces a personally salient situation” [4].

The role of affect (emotions) is considered crucial in the learning process, as well as in influencing learning itself, since it is linked to notions such as motivation, interest, and attention [5]. Earlier still, it was postulated [6] that learning most often takes place during an emotional episode; thus, the interaction of affect and learning may provide valuable insight in how people learn. Similarly, the relationship between learning and affective states is evident in other studies as well. In this way, a person’s affective condition may systematically influence how they process new knowledge. It has been reported [7] that expert teachers are able to have a positive impact on their students’ learning by recognizing and responding to their emotional states. Accordingly, attributes like curiosity, among other affective states, are identified as an indicator of motivation [8]. Such attributes constitute a driver for learning, being useful to motivated and affectively engaged learners in order to become more involved and to display less stress and anger [9-11], greater pleasure and involvement [10], and less boredom [11].

The well-established pleasure, arousal, dominance (PAD) psychological model of emotional states [12,13] represents all emotions deploying the above three dimensions on a scale from negative to positive values. Pleasure regards how pleasant (joy) or unpleasant (anger, fear) one feels about something. Arousal corresponds to how energized or bored one feels, and dominance refers to how dominant or submissive one feels. Even though the PAD model was originally configured with three components, the first two, pleasure and arousal, seem to have been used to a greater extent by researchers than dominance [14], mainly due to the fact that “all affective states arise from two fundamental neurophysiological systems, one related to valence (a pleasure–displeasure continuum) and the other to arousal” [15].

### Sensor-Based Affect Recognitions

Biosignals such as heart rate (HR), blood volume pressure, external body temperature, electrodermal activity (EDA), and electroencephalography (EEG) are the physiological signals of the human body that drastically change during changes of emotional state [16]. Analyzing the change of physiological signal recordings can determine the emotional state of the human [17] by using devices that record and track the changes of the physiological signals, called biosensors [18,19]. Analyzing the data recordings from those devices for emotional state detection has been the research interest of recent studies. EDA and EEG biosignals have been used for the detection of stress during indoor mobility [20,21]. Additionally, EEG biosignal classification for emotion detection using the valence-arousal model of affect classification was also explored by our group [22].

### Emotional Content and Brain Activation With Regards to Learning

Affective states, such as fear, anger, sadness, and joy, alter brain activity [23,24] and are associated with the neurophysiological interaction between cortical-based cognitive states and subcortical valence and arousal systems [15]. Today, ever-increasing neuroimaging data verify the importance of specific emotion-related brain regions, such as the orbitofrontal cortex, the dorsolateral prefrontal cortex, the cingulate gyrus, the hippocampus, the insula, the temporal regions, and the amygdala, in forming emotions and in the process of learning [25-32]. The hippocampus and amygdala form an apparatus of memory that houses two distinct yet functionally interacting mnemonic systems of declarative and nondeclarative memory [33,34]. Along with the other previously mentioned areas, they belong to the limbic system and the pathway of memory formation, consolidation, and learning [35], although those are wider processes that are not intrinsically tied to anatomical restraints and cannot be precisely localized [36,37].

Based on studies in animals and humans, which showed evidence of the critical role of the amygdaloid complex (AC) in emotional reactions [38-40], other studies in humans using functional magnetic resonance imaging have identified AC activation in response to affectively loaded visual stimuli [41-43]. Additionally, positron emission tomography scan studies have identified a connection between emotional stimuli and activity in the left AC [44]. The AC takes part in a system of nondeclarative memory formation and emotional conditioning using mechanisms such as long-term potentiation [45,46]. A functional asymmetry has also been identified between left and right AC, as negative emotional conditioning, especially based on fear, leads to a nondeclarative learning process, tracked predominantly in left AC [47]. Emotion valence and information encoding favor this asymmetry with regard to both positive and negative stimuli [48,49], while arousal has been linked to electroencephalographic theta waves from the amygdala [50]. This asymmetry also entails different modalities of encoded information and, while the left side has been functionally associated with language and detailed affective information, the right side has been functionally associated with imagery [49]. Moreover, it has been demonstrated that the declarative and nondeclarative systems interact during the process of learning, as emotion influences encoding by modulating the qualitative characteristics of attention, while episodic and active learning have also been proven to condition emotional response through memory formation [47,51-54].

Age difference is another factor that may potentially moderate cognitive appraisal of emotional content. Taking into consideration the age-related positivity effect, eye-tracking was used [55] to test for potential age differences in visual attention and emotional reactivity to positive and negative information between older and younger adults. It was discovered that when older adults processed negative emotional stimuli, they attended less to negative image content compared with younger adults, but they reacted with greater negative emotions. Finally, several studies have explored sex differences in the neural correlates of emotional content reactivity [41,56]. These have often highlighted the key role of the amygdala. It was found [57] that women exhibited increased activation in the amygdala, dorsal midbrain, and hippocampus. Similarly, men exhibited increased activation in the frontal pole, the anterior cingulate cortex and medial prefrontal cortex, and the mediodorsal nucleus of the thalamus.

### Technology-Enhanced Immersive Medical Education and Virtual Reality

Information and communication technologies (ICT) have shaped interventions for health care and wellness from their beginning. Digital innovations reduce costs, increase capacities to support growth and address social inequalities, and improve diagnostic efficacy and treatment effectiveness. Contemporary medical education in particular has progressed extensively towards widely diverse learning resources and health care–specific educational activities in the ICT domain [58]. The incentive behind this lies with the necessity for worldwide access to clinical skills, unconstrained by time and place [59]. This potential of ICT in medical education is multiplied by the parallel advancement of web technologies and the proliferation of interactive learning environments with immediate, content-related feedback [60].

Currently, medical education is mostly based on case-based or problem-based learning and other small-group instructional models [61,62]. These include simulations, scenario narratives and other structured, task-based learning episodes. Scenario narratives in particular, termed virtual patients (VPs) in the health care sector, are serious game episodes designed custom to the learning objectives but also aligned with the expectations and skill sets of students in order to provide a game-informed, media-saturated learning environment. In that way, students can explore a case through multiple avenues, exercise their decision-making skills, and explore the impact of those decisions in a safe but engaging way [63,64]. VPs are defined as “interactive computer simulations of real-life clinical scenarios for the purpose of healthcare and medical training, education or assessment” [65]. Web-based VPs, unlike real patients, are consistently repeatable, since they are structured as branching narratives [66], offering few limitations with respect to time, place, and failure during the practice of clinical skills. Medical students have the opportunity to practice on a diverse set of rare and difficult diseases that they may later encounter in clinical practice [67]. Finally, the reproducibility of the case outcomes and the provisions for standardized validated assessment that exist in most VP platforms have established the use of VPs as an effective and important tool for modern medical education [68-70]. Due to these advantages, there is a global trend towards increased development of VPs, with many academic institutions working towards this goal [71]. The extended impact of VPs for medical education has been recognized, and standardization solutions for repurposing, reusing, and transferability have been initiated early on [65], with a formal standard, the MedBiquitous virtual patient standard, being finalized as early as 2010 [72,73]. Contemporary improvements, such as semantic annotations, have been implemented for easy reusability of VP content [74], while other efforts have focused on various fields, like elderly care [75], and even on intensifying experiential means, like virtual worlds [76,77], virtual reality, and augmented reality [78].

On the experiential front, many ideas have been implemented. One of them is the virtual laboratory. Virtual labs use simulations and computer models, along with a multitude of other media, such as video, to replace real-life laboratory interactions. A virtual lab consists of several digital simulations supported by discussion forums and video demonstrations, or even collaboration tools and stand-alone complex simulations [79]. Such interactive environments facilitate self-directed, self-paced learning (eg, repeating content, accessing content at off hours). That way, learners maintain initiative and increased engagement in the learning process, while interactivity hones laboratory skills that go beyond simple knowledge transfer. These laboratory skills strengthen the core areas of weakness in the contemporary medical curriculum. Hands-on laboratory techniques are usually not available for training to students due to cost, time, or safety constraints [80,81]. This leaves medical students with theoretical understanding but a lack of real-world clinical and lab skills [81].

An approach readily supportive of the virtual lab that can incorporate VP serious gaming is the implementation of virtual reality (VR), augmented reality, and, recently, mixed reality (MR) technologies, especially with the advent of devices like the Microsoft HoloLens (Microsoft Corp). The distinctions are somewhat blurred at times, but virtual reality is the substitution of external sensory inputs (mainly visual and audio) with computer-generated ones using a headset device. Augmented reality is the superposition of digital content over the real world that uses either 2-dimensional (2D) or 3-dimensional (3D) markers in the real-world environment. Finally, mixed reality is similar to augmented reality, with one key difference. Instead of the real-world marker being a preprogrammed static item or image, the superposition of content is done after a 3D mapping of the current environment has been completed. This way, features can be used in intuitive ways, like 3D models positioned on tables or 2D images or notes hanging on walls. There is evidence that such technologies significantly increase the educational impact of a learning episode and can subsequently greatly affect educational outcomes [82]. Realized examples include experiential world exploration [83], physics and chemistry concept visualizations with high engagement impact [84-86], and even the incorporation of such modalities for VPs [78]. It is this immediate engagement capacity of these modalities that can not only motivate the student but also allow for internalization of the educational material and, thus, avoidance of conceptual errors [87].

### Aim and Scope of This Work

From this introduction, it becomes apparent that there is currently a sufficient body of research identifying both the impact and the capacity of digital tools for detecting and affecting the emotional state of users in their learning activities. Contemporary integrated wearable and unobtrusive sensor suites can provide objective, biosignal-based (as opposed to self-reported or inferred) emotion recognition. In addition, the proliferation and impact of immersive resources as medical education support tools provide strong motive to explore the feasibility of implementing in them real-time, evidence-based affective analytics.

Using commercial wearables and EEG sensors, this work presents the first, to the authors’ knowledge, feasibility pilot of real-time, evidence-based affective analytics in a VR/MR-enhanced medical education VP serious game.

## Methods

### Equipment, Affective, and Educational Setup

A total of 11 healthy participants took part in our study after providing written informed consent. The participants included medical students (4 participants), medical school postgraduates (3 participants), and neurosurgeons (4 participants). The participants were informed that they would take part in 2 VP scenarios. The 2 scenarios were (1) a simple emergency response VP scenario that is familiar to all medical students beyond the third year of their studies, implemented on a simple web-based platform (OpenLabyrinth), and (2) a neuroanatomy-focused case regarding the ascending and descending pathways of the central nervous system, designed for a specialized neuroanatomy lecture at the graduate/postgraduate level and implemented in the MR HoloLens platform. The choice of scenario for the MR platform (Microsoft HoloLens) was forced to be this specific resource, as it was the only one that was available in scenario-based format. Both scenarios were medical narrative games that were guided by player choice. In the case of the web-based VP game, the users chose their responses from a multiple-choice panel on each of the VP game’s pages. In the MR VP game, the users visualized the actual case through the HoloLens device, seeing and tacitly manipulating the relevant parts of the anatomy as they were described to them by narrative text. Selections in this modality were conducted by hand gestures in relevant locations of the actual anatomy that was presented to the user.

Due to the diverse background of the participants, they were exposed to the educational episodes in different ways. All groups tackled the first scenario on their own according to their knowledge. The medical students were fully guided in the MR scenario, since the educational content of it was beyond their knowledge. That means that they were free to ask any technical or medical question to the research team and they were provided with full guidance to select the correct answer. The postgraduate medical students were asked to resolve the MR scenario on their own and were offered assistance if they appeared to be stuck in and unable to proceed from a specific stage of the scenario. That means that they were free to ask any technical or medical question to the research team and they were provided with full guidance to select the correct answer. The neurosurgeons were all asked to complete the case on their own and were provided help only towards usage issues regarding the HoloLens device. That means that they were allowed to ask only technical questions about the functionality of the device.

In both scenarios, brain activity was recorded via an EEG, along with biosignals through a wearable unit. EEG signals were acquired using a 2-channel EEG amplifier (Nexus-10; Mind Media) [88] connected via Bluetooth to the PC, where signals were recorded (sampling rate 256 Hz) and preprocessed in real time. EEG electrodes were placed at the Fz and Cz positions, references at A1 and A2 (earlobes), and ground electrode at Fpz of the international 10-20 electrode placement system. Preprocessing included automatic EEG artifact removal and generation of real-time theta (4 to 8 Hz), alpha (8 to 12 Hz), and beta (13 to 21 Hz) rhythm. Moreover, the composite ratio of theta over beta was additionally generated.

For continuous, real-time physiological signals for stress detection, the E4 wearable multisensory smart wristband (Empatica Inc) was used [89]. HR and EDA were recorded with a sampling rate of 1 Hz and 4 Hz, respectively.

**Figure 1:**
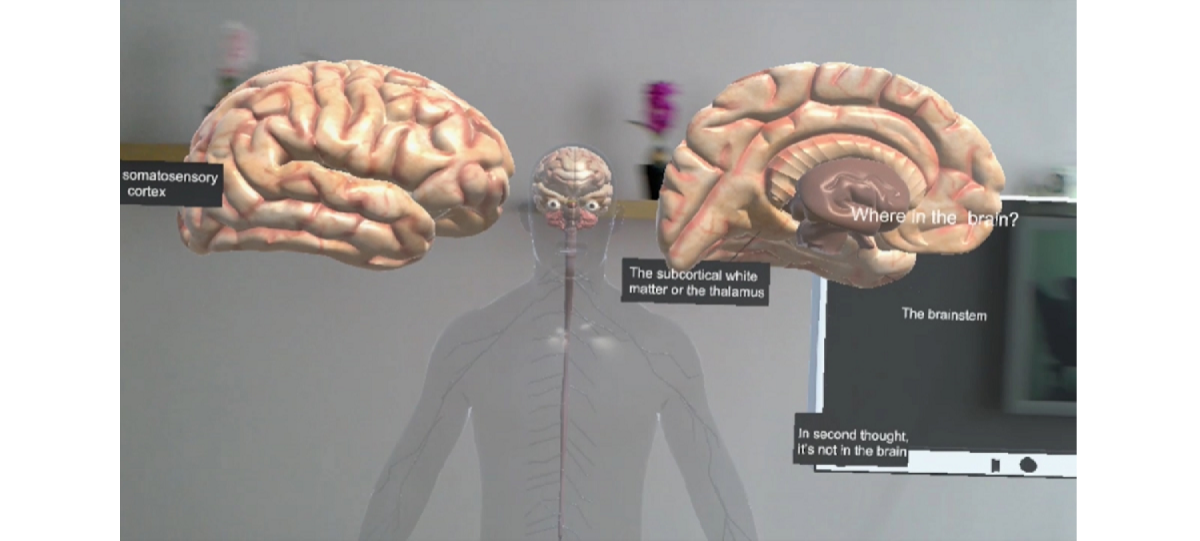
Part of the HoloAnatomy neuroanatomy virtual scenario.

In order to implement the MR component of this experiment, the Microsoft HoloLens holographic computer headset was used [90]. Microsoft HoloLens is the world's first fully untethered holographic computer, providing holographic experiences in order to empower the user in novel ways. It blends optics and sensors to deliver seamless 3D content interaction with the real world. Advanced sensors capture information about what the user is doing, as well as the environment the user is in, allowing mapping and understanding of the physical places, spaces, and things around the user. The scenario used for the experiment was an exploratory interactive tutorial on the main central nervous system pathways in the brain and spinal cord (Figure 1). For the needs of this pilot experimental setup, 2 standard PC units were also used.

All real-time signal acquisition and postprocessing were conducted in a dedicated signal acquisition and postprocessing (SAPP) PC unit, while subjective emotion self-reporting, VP educational activity, and overall time stamp synchronization was conducted through an activity and time stamp synchronization (ATS) PC unit.

In the ATS unit, the Debut video capture software (NHS Software) [91] was used to record and time-stamp all of the participant’s activities on screen for reference and manual synchronization with the internal clocks of the EEG and wearable sensor recorded from the SAPP unit.

**Figure 2:**
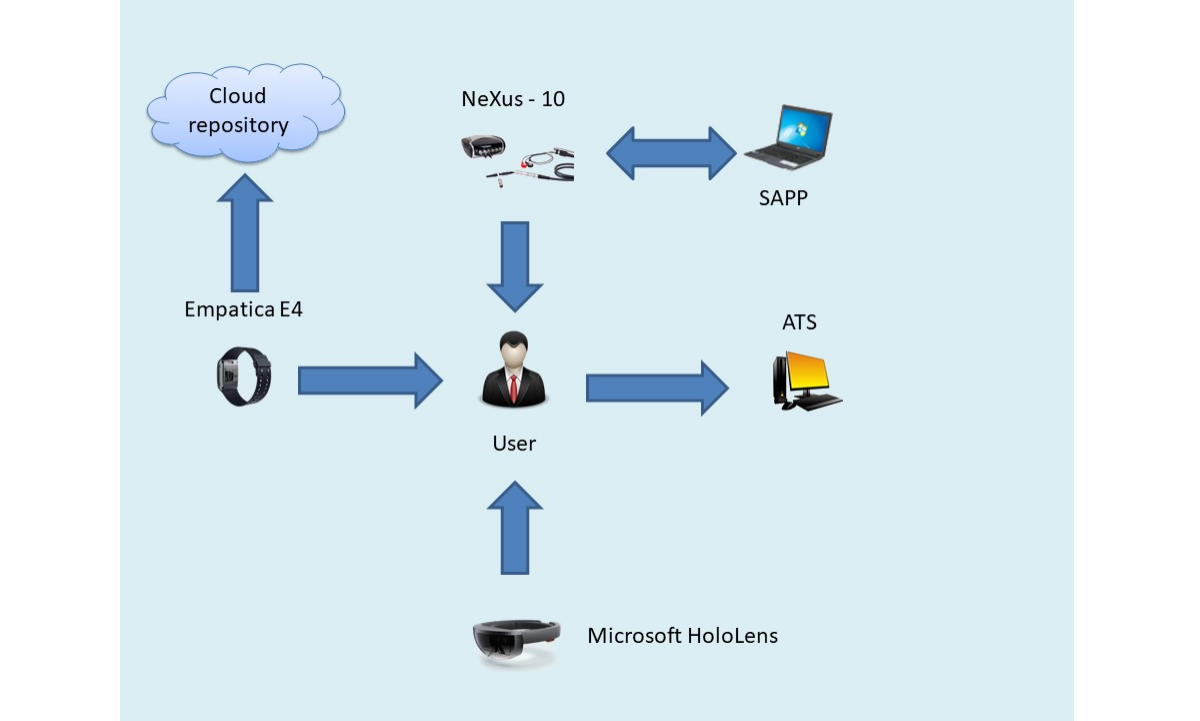
Multimodal biosignal sensors and mixed reality equipment setup. ATS: activity and time stamp synchronization; SAPP: signal acquisition and postprocessing.

The overall equipment setup and synchronization is demonstrated in Figure 2.

### Experimental Methodology

The experiment and relevant recordings took place on the premises of the Lab of Medical Physics of the Aristotle University of Thessaloniki in a quiet space. For the first session, the participant was invited to sit comfortably on a chair in front of the ATS unit’s screen, located at a distance of 60 cm. While the participant was seated, the Empatica E4 sensor was provided to them to wear and the Nexus-10 EEG electrodes were applied. The ATS unit’s screen displayed the initial page of the VP scenario and the time in Coordinated Universal Time (UTC) to allow for precise time recording. Data synchronization was conducted manually. The biosignal data acquired by the Empatica E4 and Nexus-10 devices were exported with a time stamp in UTC. Having a global time stamp both in the sensor recordings and in the educational activity computer (ATS) allowed for later offline synchronization. Specifically, the start of the time series for each sensor acquisition was synchronized in global time, together with the educational events as they were annotated manually, also in global time. The scenario is, in short, an interlinked web of pages that describes a coherent medical case.

The users navigated the interlinked web of pages by selecting their preferred answers in each part of the case from a predetermined multiple-choice list. The coordinator of the experiment was seated at a close distance next to the participant, though outside of the participant’s visual field in order to not affect participant behavior, and was operating the SAPP unit continuously overseeing the acquisition process. The recordings of this session constituted the personalized sensor baseline for this user.

For the second session, the Empatica E4 and the Nexus-10 devices were used in the same manner as in the first session. Additionally, the HoloLens holographic computer unit was worn by the subject. Sitting comfortably on their chair, the participant viewed through the holographic unit an interactive exploratory neuroanatomy tour. Interaction with the HoloLens was conducted with gestures. In order not to contaminate the EEG recordings with the motor cortex EEG responses, the gestures were conducted by the coordinator after a preset time had passed (approximately 4 seconds). The whole session was video recorded in order to facilitate activity annotation at a later time. All participants took approximately the same time to finish their session. This time was approximately 35 to 40 minutes. About 10 minutes were used for orientation and equipment placement, 10 minutes were used for the VP case, and another 15 minutes were used for the MR experience.

### Data Analysis

The acquired data (HR, EDA, alpha amplitude, theta/beta ratio) were annotated according to user activity data taken from the ATS unit and the video recording of the second session. Annotation marks in the web-based VP session were placed at the time points where the user moved to a new node in the VP scenario. These data segments of all the data sets (HR, EDA, alpha, theta/beta) formed the baseline values of each biosignal modality for this user. The acquired data of the MR VP session were annotated with marks placed at time points of the interaction gestures as they appeared in the video recording of the session. The first session segments were averaged in order to extract a global baseline average for each biosignal modality. The second session data were averaged on a per-segment basis, and the resulting data series (one data point per gesture per signal modality) were explored using descriptive statistics for quantitative differences from the baseline values.

## Results

In the conventional educational episode, the participants explored a VP scenario. As previously mentioned, the data were annotated and segmented after each user transitioned from one stage of the VP scenario to the next. Thus, after averaging all biosignal data that were annotated and segmented for these stages, we extracted the averages for the biosignals recorded in this experimental setup. These included the alpha amplitude and the theta over beta ratio for the Cz EEG position (where the sensor was placed), as well as HR and EDA values. Representative results for one participant are summarized in Table 1.

A similar process was followed for the second session, where the VR/MR neuroanatomy resource was explored by the user. The time series of the biosignals were annotated and segmented on the time stamps corresponding to each gesture-based transition that the user experienced in this resource.

**Figure 3:**
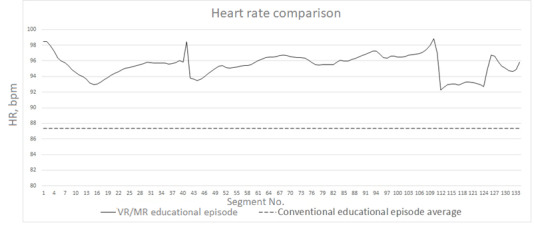
Representative heart rate segment plot for the VR/MR educational episode. Dashed line denotes the baseline established in the first experimental session. HR: heart rate; MR: mixed reality; VR: virtual reality.

**Figure 4:**
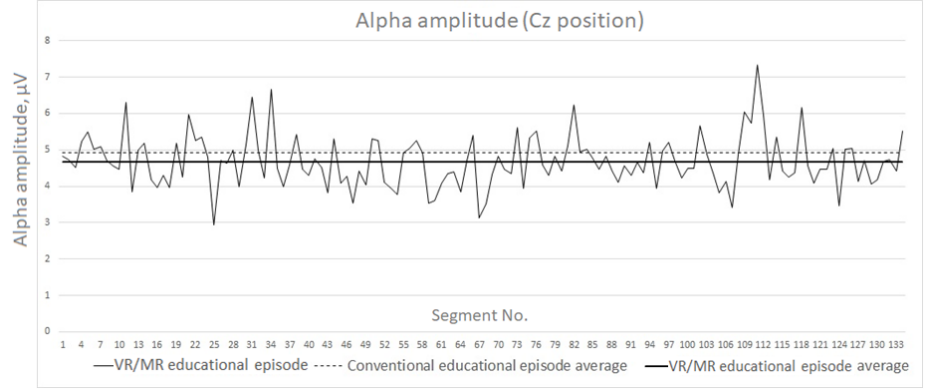
Alpha amplitude (μV) segment plot for the VR/MR educational episode for the Cz position. MR: mixed reality; VR: virtual reality.

For each segment and for the HR and EDA, average values were recorded along with the segment number. Example plots are presented for a representative participant in Figure 3 and Figure 4. For reference purposes, the baseline average was also plotted as a constant in these graphs.

**Figure 5:**
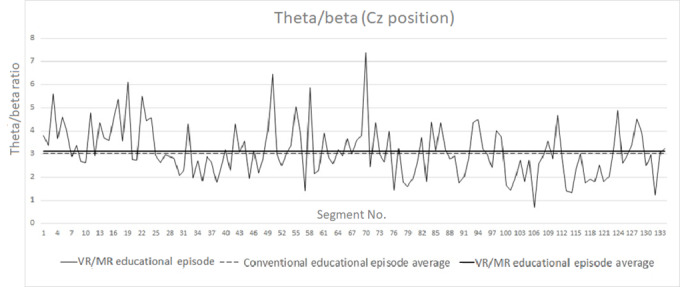
Theta/beta ratio segment plot for the VR/MR educational episode for the Cz position. MR: mixed reality; VR: virtual reality.

The same process was followed for the alpha rhythm and theta/beta ratio for the Cz point (sensor positions). Similar plots of the rhythms and ratios are presented for a representative participant in Figure 4 and Figure 5.

In Figure 4 and Figure 5, we have also included, as a plotted line, the average of the recorded signal in order to reveal even nondefinitive increases or decreases between the two experimental sessions. A representative value set is summarized in Table 2.

This analysis was performed for all 11 participants. The averaged results for each participant are presented in Table 3. Given the differentiation of each group’s affective and educational setup, the participants are also partitioned according to group in this table. Table 4 presents a per-group average for all metrics recorded in the web-based VP and MR scenarios. Given that some of these metrics (eg, EDA) are highly varied across the population, which makes averaging irrelevant for any useful purpose (see standard deviations for EDA in Table 4), Table 5 presents the average value shifts between the two scenarios. Specifically, we present the total and per-group number of participants who had increased theta/beta ratios, decreased alpha rhythm amplitude, increased HR, and increased EDA in the MR scenario compared with the web-based VP scenario.

**Table 1:** Biosignal baseline averages of a representative participant (neurosurgeon) during the conventional educational episode.

Segment	Alpha, mean (SD), μV	Theta/beta power ratio, mean (SD)	HR^a^, mean (SD), bpm	EDA^b^, mean (SD), μS
Segment 1	4.886 (0.1822)	3.198 (0.5675)	86 (2)	0.227 (0.0114)
Segment 2	5.259 (0.1822)	2.235 (0.5675)	90 (2)	0.209 (0.0114)
Segment 3	4.883 (0.1822)	3.047 (0.5675)	88 (2)	0.203 (0.0114)
Segment 4	4.921 (0.1822)	3.584 (0.5675)	89 (2)	0.203 (0.0114)
Average	4.987 (0.1822)	3.016 (0.5675)	87 (2)	0.215 (0.0114)

^a^HR: heart rate.

^b^EDA: electrodermal activity.

**Table 2:** Biosignal average of a representative participant (neurosurgeon) during the virtual reality/mixed reality educational episode.

	Alpha, mean (SD), μV	Theta/beta power ratio, mean (SD)	HR^a^, mean (SD), bpm	EDA^b^, mean (SD), μS
Average	4.680 (0.711)	3.131 (1.137)	95 (1)	3.475 (0.865)

^a^HR: heart rate.

^b^EDA: electrodermal activity.

**Table 3:** Biosignal averages per participant for the virtual reality/mixed reality educational episode.

Participant No.		Amplitude theta/beta (Cz), mean, μV		Amplitude alpha (Cz), mean, μV		HR^a^, mean, bpm		EDA^b^, mean, μS	
		VP^c^	MR^d^	VP	MR	VP	MR	VP	MR
**Students**									
	1	4.315	4.417	8.024	8.540	97	96	3.733	6.479
	2	2.734	1.788	10.228	12.443	92	92	0.525	2.438
	3	4.306	3.425	5.794	5.529	66	65	0.268	0.382
	4	2.611	3.284	7.040	7.157	96	90	0.265	7.090
**Neurosurgeons**									
	1	3.016	3.131	7.033	7.146	87	95	0.215	3.475
	2	1.438	1.598	6.023	6.223	88	82	5.788	14.539
	3	1.986	1.679	6.396	8.072	75	76	1.019	3.076
	4	3.935	5.420	10.725	9.606	73	79	0.538	0.540
**Postgraduates**									
	1	2.439	2.532	7.831	7.801	75	70	0.371	0.307
	2	2.580	3.331	20.523	13.933	90	84	0.387	4.516
	3	1.971	1.823	7.163	6.926	77	79	1.459	2.671

^a^HR: heart rate.

^b^EDA: electrodermal activity.

^c^VP: virtual patient.

^d^MR: mixed reality.

**Table 4:** Biosignal averages per group for the virtual reality/mixed reality educational episode.

Group	Amplitude theta/beta power ratio (Cz), mean (SD)		Amplitude alpha (Cz), mean (SD), μV		HR^a^, mean (SD), bpm			EDA^b^, mean (SD), μS	
	VP^c^	MR^d^	VP	MR	VP	MR	VP		MR
Students	3.49 (0.82)	3.23 (0.94)	7.77 (1.62)	8.42 (2.56)	87 (13)	86 (12)	1.198 (1.467)		4.097 (2.79)
Neurosurgeons	2.59 (0.96)	2.90 (1.78)	7.03 (2.19)	7.15 (1.86)	81 (7)	83 (7)	1.890 (2.269)		5.407 (5.391)
Postgraduates	2.33 (0.26)	2.56 (0.62)	11.84 (6.15)	9.55 (3.12)	81 (7)	77 (6)	0.739 (0.509)		2.498 (1.72)

^a^HR: heart rate.

^b^EDA: electrodermal activity.

^c^VP: virtual patient.

^d^MR: mixed reality.

**Table 5:** Biosignal average value shifts for the virtual reality/mixed reality educational episode.

Group	Increased amplitude theta/beta power ratio (Cz), n	Decreased amplitude alpha (Cz), n, μV	Increased HR^a^, n, bpm	Increased EDA^b^, n, μS
Total	6	5	2	9
Students	2	1	0	4
Neurosurgeons	3	1	3	4
Postgraduates	2	3	0	2

^a^HR: heart rate.

^b^EDA: electrodermal activity.

## Discussion

### Originality

This work presented a pilot study for technically achieving the capacity to obtain evidence-based, real-time affective analytics from users of a VR/MR educational serious game resource. Three participant groups were included, namely undergraduate medical students, medical school postgraduates, and neurosurgeons. This is the first time that a multitude of recording and interaction devices were integrated within a cohesive and contemporary educational episode. This integration led to a plausible neurophysiological interpretation of recorded biosignals regarding engagement and other affective analytics metrics.

The technical barriers for this endeavor were significant. While the wrist-wearable sensor is unobtrusive and easy to wear, obtaining EEG recordings while simultaneously wearing a highly sophisticated electronic device like the Microsoft HoloLens is not easily completed. The literature has significant findings regarding EEG and VR, but usually these involve virtual environments (3D environments projected on a 2D screen) instead of true immersive virtual reality, which requires a dedicated headset [92]. In other cases, successful endeavors incorporating EEG to VR scenarios require expensive and cumbersome simulation rooms [93]. This work is the first, to the authors’ knowledge, that involves (1) the first wearable, truly immersive mixed reality holographic computer (the MS HoloLens) and (2) real-time concurrent EEG recordings in an unconfined, free-roaming setting easily transferrable to real-world educational settings.

### Principal Neurophysiological Results

Our neurophysiological investigation focuses primarily on the theta/beta ratio (the ratio of powers of theta rhythm to beta rhythm) and the amplitude of alpha rhythm (8 to 12 Hz). An increase of theta (4 to 8 Hz) power in EEG recordings has been documented, corresponding to neurophysiological processes that facilitate both working memory and episodic memory, as well as the encoding of new information [94,95]. Moreover, when recorded at the area over midline brain regions, theta activity is also related to cognitive processes that involve concentration, sustained attention, and creativity [96-100]. In line with this, higher theta activity has been reported in the frontal-midline regions during a task of high cognitive demand, a task with increasing working memory needs [101,102], or even a high-attention process [103-105]. On the other hand, engagement in attention-demanding tasks or judgment calls is reported to lead to alpha power suppression [106,107].

Our results, in the context of the educational and affective setup of the experiment, agree with the previously reported literature.

The undergraduate student group presented a decrease in theta/beta ratio activity and an increase in alpha rhythm amplitude. These results can be interpreted as engagement in high-demand cognitive functions while under suppression of judgement calls. As we described in the “Equipment, Affective, and Educational Setup” section, undergraduate students were offered the correct choices by the researchers during the MR scenario, given its very demanding neuroanatomy content. In that context, undergraduate students concentrated on the mechanical tasks of using the MR equipment, while no judgement calls were made by them (it can be said that cognitive control of the scenario was relegated to the researcher facilitating the student).

The second group, the postgraduate students, presented an increase in theta/beta ratio activity. They also presented a decrease in alpha rhythm amplitude during the MR serious game scenario. As we previously described, these students were asked to individually solve a very challenging (for their education level) medical scenario. Thus, they had to use all their cognitive faculties in order to overcome this challenge. The results are consistent with the educational setup for this group.

The third group, consisting of specialist neurosurgeon doctors, presented an increase in theta/beta ratio. They also presented an increase in alpha rhythm amplitude, similar to the undergraduate medical students. In this case, the participants (neurosurgeons) were presented with a scenario that required their concentration and initiative to solve, but they were not seriously challenged, since the educational material covered in the case was well within their skills. Thus, they had to commit cognitively to the task, especially to use the MR equipment, but not fully. This cognitive engagement ambivalence is demonstrated by the concurrent increase in theta/beta ratios and alpha rhythm amplitudes.

Regarding the EDA and HR results, these value were significantly elevated in the VR/MR session versus the baseline educational episode. Elevated HR and EDA are established signs of high arousal, independent of valence [108,109]. HR remained more or less steady on average in all groups, a fact that can be attributed to the overall relaxed environment of the experiment (seated participation, silent room, etc) and the resilience of the average HR in short-term variations. However, almost all the participants presented a significant increase in EDA, which can be attributed to the overall novelty factor of wearing a sense-altering digital device, as well as the immersion and excitement of the interaction with the VR/MR educational resource.

### Limitations

Despite the overall promising results, this work contains some inherent limitations, mostly linked with the novelty of its aim and scope. A significant limitation is the small and diverse group of subjects that were used for the pilot run of the multimodal signal acquisition configuration. It must be emphasized that given the sampling rate of the biosensors, every 1 minute of recording provided 15,360 samples (256 Hz × 60 s) of EEG per channel, 60 samples (1 Hz × 60 s) of HR, and 240 samples (4 Hz × 60 s) of EDA. This high-density data throughout the study allowed for rather definitive biosignal results to be extracted on a per-participant basis. While the extensive data set gathered from participants provided credible results for this feasibility study, obviously there is the need to expand the participant sample in order to explore personalization and statistical verification challenges.

Another core limitation of this study is the low affective impact of the MR case. Furthermore, all patients experienced the educational content only once. The case had significant immersive content, including animation and audio cues as rewards and motivations for the user. However, it lacked impact and significant narrative consequences for the users’ actions.

Thus, future work will require a larger user base, as well as more frequent exposure to emotional affective educational stimuli with emotional and narrative specificity of this suite in a VR/MR approach. Specific emotion-inducing content needs to be implemented in this modality in order to assess the biosignal variations as the emotional content clashes (or cooperates) with the VR/MR platform’s inherently high arousal and emotional impact (for example, error-based training). Finally, as previously mentioned, a future goal to pursue based on this work is the integration of this biosensor suite in an intelligent tutoring system (ITS) for immediate medical teaching support that includes VR/MR resources.

### Comparison With Prior Work

Similar endeavors for affective analytics in non–technology-heavy medical education episodes have already been initially explored [110]. However, this work is the first proof of application of at least one configuration that is realistically unconfined and applicable in a simple educational setting for emotion detection in a technology-heavy VR/MR-based medical education episode. The full scope of application for this work is the integration of sensors and devices for incorporating objective, sensor-based affective analytics in VR/MR educational resources provided by ITSs.

ITSs are computer systems that aim to provide immediate and customized instruction or feedback to learners [80], usually without requiring intervention from a human teacher. ITSs typically aim to replicate the demonstrated benefits of one-to-one (personalized) tutoring to one-to-many instruction from a single teacher (eg, classroom lectures) or no teacher at all (eg, online homework) [111]. An ITS implementation is based on students’ characteristics and needs, and it analyzes and anticipates their affective responses and behaviors in order to allow more efficient collection of information of a student’s performance, handle content adjustment, tailor tutoring instructions upon inferences on strengths and weaknesses, suggest additional work, and in general improve the learning level of students [112].

In that context, the integration of emotion detection for content and experience customization is not a new endeavor. Affective tutoring systems use bias-free physiological expressions of emotion (facial expressions, eye-tracking, EEG, biosensors, etc) in order “to detect and analyze the emotional state of a learner and respond in a way similar to human tutors” [113], hence being able to adapt to the affective state of students [114]. The results of this work provide initial evidence for the integration of multisensor configurations with a VR/MR platform and the synchronization and coordination of such a suite by an affect-aware ITS.

### Conclusions

VR/MR is a versatile educational modality, especially in the highly sensitive medical domain. It provides the capacity both for highly impactful narrative, which is crucial for making doctors invested in the educational process, and for building decision-making skills and competences. It also provides the ability, through immersive simulation, for the medical learner to practice manual skills in surgical specialties. This dual capacity makes this modality highly impactful and sought after in the medical field. Therefore, the capacity to provide personalized, context-specific content and feedback based on the learner’s emotional state is crucial in this modality for both self-directed and standard guided medical learning. This work provides the first proof of application for such endeavors.

## Abbreviations

2D: 2-dimensional
3D: 3-dimensional
AC: amygdaloid complex
ATS: activity and time stamp synchronization
EDA: electrodermal activity
EEG: electroencephalography
HR: heart rate
ICT: information and communication technologies
ITS: intelligent tutoring system
MR: mixed reality
PAD: pleasure, arousal, dominance
SAPP: signal acquisition and postprocessing
UTC: Coordinated Universal Time
VP: virtual patient
VR: virtual reality
